# Mobility and Muscle Strength Trajectories: The Effect of Mediterranean Diet, Physical Activity, and Social Support

**DOI:** 10.1093/geroni/igab046.274

**Published:** 2021-12-17

**Authors:** Marguerita Saadeh, Federica Prinelli, Davide Vetrano, Weili Xu, Anna-Karin Welmer, Serhiy Dekhtyar, Laura Fratiglioni, Amaia Calderón-Larrañaga

**Affiliations:** 1 Aging Research Center, Karolinska Institutet, Stockholm, Stockholms Lan, Sweden; 2 Aging Research Center, Dept NVS, Karolinska Institutet, Neurobiology, Care Sciences and Society, Stockholms Lan, Sweden; 3 Karolinska Institutet, Solna, Stockholms Lan, Sweden; 4 Karolinska Institutet, 17175, Stockholms Lan, Sweden

## Abstract

Decline in physical function varies substantially across older individuals due to several biological and extrinsic factors. We aimed to determine the effect of modifiable factors -such as dietary patterns, physical activity and social support- and their interaction with mobility and muscle strength decline after age 60. We analyzed data from 1686 individuals aged 60+ from the population-based Swedish National study on Aging and Care in Kungsholmen. The Mediterranean Diet Score was calculated based on a validated food frequency questionnaire. Physical activity was categorized based on current recommendations, and social support was measured according to participants' perceived material and psychological support. Participants’ physical function was assessed over 12 years through changes in walking speed (m/s) and chair stand time (s). Linear mixed models adjusted for socio-demographic and clinical factors were used. Subjects with high adherence to Mediterranean diet were <78 years (82.3%), women (56.1%), married (61.1%), with university education (52.8%), high levels of social support (39.3%) and health-enhancing physical activity (51.5%). One-point (over nine) increase in the MDS was associated with a slower annual worsening in walking speed (β*time=0.001; p=0.024) and chair stand time (β*time=-0.014; p=0.008). The protective effect of Mediterranean diet was highest among subjects reporting high social support (β*time=-0.065, p=0.026 for chair stands) and high physical activity (β*time=0.010, p=0.001 for walking speed), beyond the effect of each exposure individually. A higher adherence to Mediterranean diet, especially in combination with recommended levels of physical activity and high social support, contribute to delay the decline in physical function observed with aging.

